# Prevention of laparoscopic surgery induced hypothermia with warmed humidified insufflation: Is the experimental combination of a warming blanket synergistic?

**DOI:** 10.1371/journal.pone.0199369

**Published:** 2018-07-11

**Authors:** Eric Noll, Sophie Diemunsch, Julien Pottecher, Jean-Pierre Rameaux, Michele Diana, Eric Sauleau, Kurt Ruetzler, Pierre Diemunsch

**Affiliations:** 1 Service d’Anesthésie Réanimation Chirurgicale, Hôpitaux Universitaires, Strasbourg, France; 2 Institut Hospitalo-Universitaire de Strasbourg, Strasbourg, France; 3 Département de Bio statistique, CHU Strasbourg, Strasbourg, France; 4 Department of Outcomes Research, Anesthesiology Institute, Cleveland Clinic, Cleveland, United States of America; Public Library of Science, UNITED KINGDOM

## Abstract

**Introduction:**

Maintaining normothermia during anesthesia is imperative to provide quality patient care and to prevent adverse outcomes. Prolonged laparoscopic procedures have been identified as a potential risk factor for hypothermia, due to continuous insufflation of cold and dry carbon dioxide. Perioperative hypothermia is associated with increased hospital cost and many complications including; impaired drug metabolism, impaired immune function, cardiac morbidity, shivering, coagulopathy.

**Methods:**

In this experimental study, four pigs underwent four interventions each, resulting in 16 total trials. Using standardized general anesthesia in a randomized Latin-square sequence the four interventions include: 1. Control group without an administered pneumoperitoneum, 2. Administered standard pneumoperitoneum using 21°C insufflated gas and under-body forced-air warming, 3. Administered pneumoperitoneum with insufflation of warmed/humidified carbon dioxide, 4. Administered pneumoperitoneum with insufflation of warmed/humidified carbon dioxide and under-body forced-air warming. The primary outcome was distal esophageal temperature change 4 hours after trocar insertion.

**Results:**

Four hours after trocar insertion, pigs in the control group lost 2.1 ± 0.4°C; pigs with warmed and humidified insufflation lost 1.8 ± 0.4°C; pigs with forced-air warming group lost 1.3 ± 0.9°C; and pigs exposed to a combination of warmed and humidified insufflation with forced-air warming increased by 0.3 ± 0.2°C.

**Conclusion:**

This experimental animal study provides evidence that a combination of warmed and humidified insufflation of carbon dioxide (CO_2_) in conjunction with forced-air warming is an effective strategy in the prevention of perioperative hypothermia. Further clinical trials investigating humans are therefore indicated.

## Introduction

Perioperative hypothermia is associated with adverse outcomes including impaired drug metabolism, impaired immune function, cardiac morbidity, shivering, coagulopathy, and increased use of hospital resources [1–5]. Several methods have been developed for maintaining normothermia during surgery. A few normothermia methods include warming patients before the induction of anesthesia [6–8]; conductive warming by circulating water garments or water mattresses; covering the patient’s back and/or other parts of the body [9, 10]; highly efficient “energy transfer” pads [11, 12]; and convective warming from forced-air blanket systems [13].

Convective (forced-air) warming is the most common intraoperative warming strategy since it is safe, easy to use, and has the potential to transfer a considerable amount of heat to the anterior surface of patients. There are limitations to the use of forced-air warming however. Forced-air warming is relatively inefficient on a surface-area to volume basis and it is often not possible, or practical, for warming patients having large open-procedures. It is known that single forced-air warming does not reliably prevent perioperative hypothermia [14]. Due to forced-air strategy inadequacy, additional (re)-warming strategies are indicated to maintain normothermia.

Laparoscopic procedures are another risk factor for developing perioperative hypothermia due to prolonged surgical time and increased heat loss via exposure to cold/dry CO_2_ insufflation during pneumoperitoneum [15–17]. Insufflated CO_2_ is typically administered with a temperature of 21°C, which is significantly colder than the patient’s normal temperature range of 36.5–37.5°C.

Prior experiments indicate that heating and humidification of insufflated carbon dioxide may be beneficial in preventing perioperative hypothermia [18]. Furthermore, the interaction between the temperature of insufflated air and forced air warming has yet to be quantified.

We therefore conducted a randomized crossover animal trial to test the applicability and efficacy of using heated/humidified carbon dioxide that is insufflated during abdominal pneumoperitoneum for normothermia maintenance. The primary analysis was to determine if insufflating heated/humidified carbon dioxide combined with underbody forced-air warming is advantageous in maintaining normothermia during pneumoperitoneum when compared to the use of either warming strategy alone.

## Methods

After the institutional animal ethics committee approval (Ircad Committee of Ethics: ICOMETH, President Prof. Didier Mutter, approval N° 38.2012.01.041), four large white pigs aged between 2 and 3 months were included in a randomized, crossover study. All animals used in the study were managed in accordance with the French laws for animal use and care. The method of care was also in compliance with the directives of the European Community Council (2010/63/EU) with respect to the principles of 3R (Replacement, Reduction, Refinement).

Each of the four treatment days were separated by eight resting (non-intervention) days. On each study day, the four pigs were randomly assigned to one of the four interventions. Each animal underwent all of the following treatments one time:

1. General anesthesia without administered pneumoperitoneum (**control group**)
2. Under-body forced air warming blanket at 38°C (Bair Hugger) and a standard insufflation with non-humidified, non-heated CO_2_ (**forced air group**)
3. Insufflation with humidified and heated CO_2_ using the Humigard device (Fisher and Paykel Healthcare) without warming blanket (**warmed insufflation group**)
4. Combination of an under-body forced air warming blanket and insufflation of humidified and heated carbon dioxide using the Humigard device (**combination group**)

Based on the experimental approach and lack of available data from previous studies, we decided to include four pigs undergoing four interventions each, resulting in 16 interventions total. This cross-over study design is appropriate in order to demonstrate feasibility and practical implementation of the study setting. This trial may provide appropriate evidence for subsequent clinical trials.

### Protocol

Four large, male, white pigs aged between 2 and 3 months were provided by a local farming company (Copvial, Brumath, France). The pigs were housed in individual stable boxes under the supervision of an animal keeper.

The pigs were given a three day habituation period at the research facility with *ad libitum* access to food and water. On the intervention days, each of the four pigs was sedated using intramuscular administration of ketamine (20 mg/kg) and azaperone (2 mg/kg) [19]. After weighing the pigs and placing them in the supine position on the operating table, an IV catheter (22 Gauge) was inserted into the auricular vein and a crystalloid infusion was administered at a rate of 4 ml/kg/h. General anesthesia was induced with 3mg/kg propofol and 0.6 mg/kg rocuronium intravenously. After tracheal intubation (Portex Blue Line 6-mm), anesthesia was maintained with 1% end-tidal concentration of isoflurane combined with 60% nitrous oxide in oxygen. Mechanical ventilation using a semi-closed circle system (Aysis Carestation, GE Healthcare, United Kingdom or Datex Ohmeda Aespire, GE Healthcare, United Kingdom) was adjusted to maintain end-tidal PCO_2_ between 35 and 40 mmHg. Gas flow was maintained at a total of 1 Liter per hour. A temperature probe (Odam Physiogard SM 785TM) was inserted into the distal esophagus. The ambient temperature of the room was maintained near 20°C.

With the exception of the control group, a pneumoperitoneum was created by the surgeon using a Veress needle. The pigs were inflated with CO_2_ prior to the insertion of two separate laparoscopic trocars. A camera was introduced via one trocar, while the other was used to create a controlled gas leakage at 120 L/h. The treatment groups underwent a 4-hour intervention with continuous CO_2_ insufflation to maintain an intra-abdominal pressure of 10 mmHg (Thermoflator, Karl Storz, Tübingen, Germany). In the warmed insufflation group and in the combination group, the insufflated CO_2_ was humidified to saturation and heated to 37°C using a Humigard device (Fisher and Paykel Healthcare, New Zealand).

An under-body warming blanket was positioned under the pigs for the forced air and combination groups. The forced-air blower was set to 38°C, and activated immediately after tracheal intubation.

At the end of the four hour study period, the pneumoperitoneum was exsufflated and each abdominal access port was infiltrated with 5mL of 2% lidocaine (Lidocaine B. Braun, Germany). Once adequate spontaneous breathing was established, the pigs were extubated and returned to the care of the animal keeper at a facility with ad libitum access to food and water.

At the end of the fourth intervention day, data collection was complete and the animals were euthanized using deep anesthesia for 15 minutes (isoflurane 5 vol % in O_2_/N_2_O: 40%/60%) followed by a 20 mL IV injection of a saturated solution of potassium chloride.

### Measurements

Demographic and morphometric characteristics of the pigs were recorded. Time recording started after complete trocar insertion. Distal esophageal temperature and total volume of insufflated CO_2_ was recorded in fifteen minutes intervals.

### Data analysis

The temperature values are presented as mean ± standard error. Linear mixed models were used to compare the temperature for each of the 15 minutes intervals for the four intervention groups. A random subject effect was systematically included as a normal distribution with zero mean and little variance. For time effect, we used dummy variables or linear slope. For multiple comparisons adjustment, we the controlled family-wise error rate simultaneous inference, with a global 5% alpha error risk.[20] All raw data are publicly accessible in **S1 Appendix temperatures per pig and intervention.**

## Results

**Fig 1:**
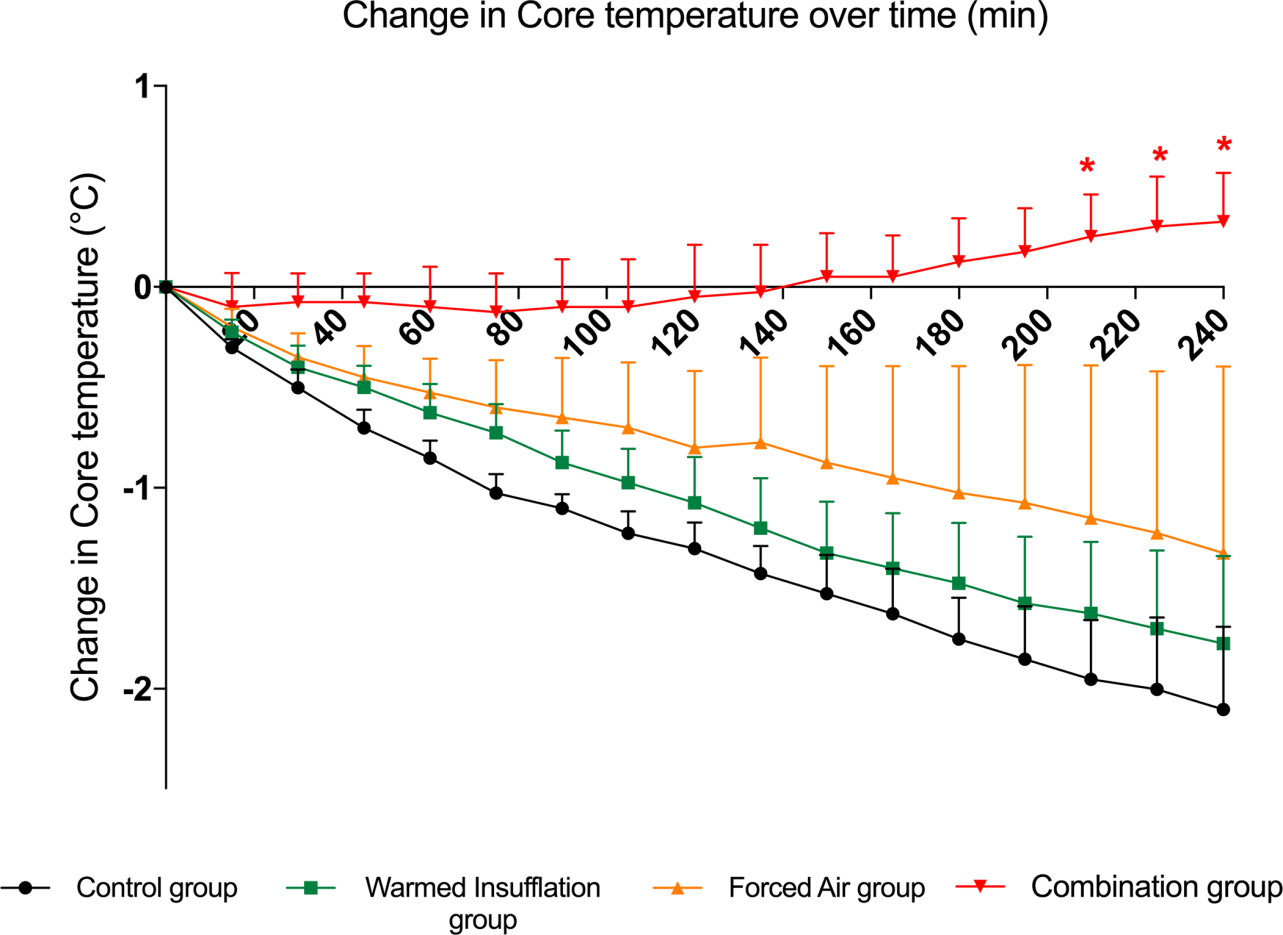
Changes in core temperatures during the study period in each group. *Circle blue line*: control group (no insufflation); *Square green line*: warmed insufflation group (heating and humidification of the insufflation CO_2_); *Triangle orange line*: forced air group (standard insufflation with lower-body warming blanket); *Triangle red line*: combination group (heating and humidification of the insufflation CO_2_ in association with underbody warming blanket).Red Star stands at time after which the difference in temperature becomes significant across groups control group versus combination group i.e. 210 min.

All four pigs underwent and survived each of the four interventions. The mean body weight was 20.9 ± 0.6 kg. The mean ambient temperature in the operating room was 20.6 ± 0.4° C. Initial group mean temperature did not statistically differ between groups (control: 36.5 ± 0.3°C; warmed insufflation: 37.1 ± 0.8°C; forced air: 36.9 ± 0.4°C; combination: 37.4 ± 0.5°C). Four hours after each intervention, the mean distal esophageal temperature dropped 2.1 ± 0.4°C in the control group, dropped 1.3 ± 0.9°C in the forced air group, dropped 1.8 ± 0.4°C in the warming insufflation group, and raised 0.3 ± 0.2°C in the combination group (**S1 Appendix temperatures per pig and intervention).** Variation in distal esophageal temperature over the course of the procedure for each group is represented in Fig 1. Considering the entire experimental time course, the slope in core temperature is not statistically significant in the warming insufflation plus forced-air group (combination group). The observed decrease in distal esophageal temperature was statistically significant in the control group when compared to the combination group. Moreover, statistically significant differences time slope differences were noted between the control group and the combination group, as well as between the warmed insufflation and combination group.

When the core temperature was divided into categorical values with time, the difference in core temperature between the control and combination group was found to be statistically significant with 210 min of insufflation.

## Discussion

This study demonstrates the potential advantage of using a forced-air warming blanket in combination with insufflating heated/humidified CO_2_ for normothermia maintenance in pigs undergoing pneumoperitoneum in comparison to prior techniques.

Hypothermia during anesthesia is common among surgical patients, thus warming is necessary. Even with current warming application, the core temperature typically drops during the first 60 to 90 minutes of surgery. This drop in temperature is mainly attributed to the initial core heat redistribution to the peripheries [21, 22]. After the redistribution period, general anesthesia causes a further decrease in temperature due to peripheral vasodilatation and increased peripheral blood flow [22, 23]. Similar to the clinical setting, the pigs core temperature dropped over the time, as demonstrated in our control group.

Forced-air warming is intended to prevent heat loss by covering the maximum amount of skin-surface area [24]. While normothermia was better maintained by forced-air warming compared to the control group, it still eventually failed in the prevention of inadvertent perioperative hypothermia.

**Heating and humidification of insufflated carbon dioxide alone** was not sufficient for temperature maintenance as the pigs became hypothermic over time. These findings were not surprising, as the humidification and heating of insufflated CO_2_ only compensates for the heat loss due to using the standard cold and dry CO_2_ insufflation of the peritoneum. However, this method does not prevent other heat loss mechanisms from occurring, such as peripheral vasodilatation [18]. Nevertheless, the effect on heat loss prevention was unimpressive when compared to the control group.

Interestingly, the reduction of core temperature in this group (1.8 ± 0.4°C) is similar to that previously reported in another study (1.9 ± 0.1°C), though the insufflation gas flow differed significantly (180 L/h vs. 120 L/h in the current study) [1818]. As a consequence of this, insufflation gas flow per hour may be important in high-flow settings. The gas flow was sufficient in preventing specific heat loss induced by peritoneal insufflation, but again was not effective in stopping heat loss based on peripheral vasodilatation. Therefore, further investigation to compare moderate to low flow insufflation in a clinical setting is warranted.

Both heating/humidification of insufflated CO_2_ and forced-air warming techniques failed to prevent inadvertent perioperative hypothermia when used separately. It seems reasonable that the combination of the two techniques might be beneficial, as they are acting on different thermo-physical heat loss pathways in separate anatomical areas. It should be noted that this combination approach was not able to prevent redistribution, but the effect was advantageous in comparison to the other groups. This was not surprising since only pre-warming may prevent redistribution.

This study has several limitations. First, this study intended to provide initial experimental data and was designed as a pilot study for subsequent clinical trials. Increased heat loss due to the insufflation of cold CO_2_ is known, and used as a part of the basis for this trial[1818]. Based on ethical considerations and the aim to reduce the number of animals used, we did not include a fifth intervention consisting of standard cold insufflation without any warming strategy applied. While the overall number of animals studied and interventions performed is low, is was sufficient enough to provide reliable initial data for the indication for future clinical trials. Administered fluids were not warmed, which is common practice in the clinical setting for human patients. The capacity of warming fluids prior to administration to increase the core temperature is limited. An advantage may be the prevention of further decreasing the core temperature by administering warm fluids rather than cold fluids [22]. Finally, since this study was performed using animal subjects, our findings cannot be translated directly into daily clinical practice. The results obtained by this study appear reliable and may serve as a basis for subsequent trials involving humans.

## Conclusion

This experimental study showed that neither the warmed/humidified CO_2_ insufflation, nor the forced-air warming blanket alone were effective in the prevention of perioperative hypothermia. This trial indicated better maintenance of temperature in combining the two approaches since they act on different anatomical areas and use different thermo-physical concepts. A human clinic trail evaluating the prevention of inadvertent perioperative hypothermia in long lasting laparoscopic cases is needed.

## Supporting information

S1 Appendix Temperatures per pig and intervention. (PDF)
