# Supplementary material for: Prevention of laparoscopic surgery induced hypothermia with warmed humidified insufflation: Is the experimental combination of a warming blanket synergistic?
Source: PLoS One. 2018 Jul 11;13(7):e0199369. doi: 10.1371/journal.pone.0199369 (PMC6040690; doi:10.1371/journal.pone.0199369)
Supplement: S1 Appendix — (PDF) [file pone.0199369.s001.pdf]

pig 4	control group		
temps	tempera	volume	deltaT
0	35.9	0	0
15	35.6	0	-0.3
30	35.6	0	-0.3
45	35.4	0	-0.5
60	35.2	0	-0.7
75	35	0	-0.9
90	34.8	0	-1.1
105	34.6	0	-1.3
120	34.5	0	-1.4
135	34.3	0	-1.6
150	34.1	0	-1.8
165	33.9	0	-2
180	33.8	0	-2.1
195	33.6	0	-2.3
210	33.4	0	-2.5
225	33.2	0	-2.7
240	33	0	-2.9

pig 1	control group		
temps	tempera	volume	deltaT
0	37.1	0	0
15	36.9	0	-0.2
30	36.7	0	-0.4
45	36.5	0	-0.6
60	36.3	0	-0.8
75	36.1	0	-1
90	36.1	0	-1
105	36.1	0	-1
120	36.1	0	-1
135	36	0	-1.1
150	36	0	-1.1
165	36	0	-1.1
180	35.8	0	-1.3
195	35.8	0	-1.3
210	35.8	0	-1.3
225	35.9	0	-1.2
240	35.9	0	-1.2

pig 2	control group		
temps	tempera	volume	deltaT
0	36.2	0	0
15	35.8	0	-0.4
30	35.6	0	-0.6
45	35.4	0	-0.8
60	35.4	0	-0.8
75	35.3	0	-0.9
90	35.2	0	-1
105	35.1	0	-1.1
120	35	0	-1.2
135	34.9	0	-1.3
150	34.9	0	-1.3
165	34.8	0	-1.4
180	34.7	0	-1.5
195	34.7	0	-1.5
210	34.6	0	-1.6
225	34.6	0	-1.6
240	34.6	0	-1.6

pig 3	control group		
temps	tempera	volume	deltaT
0	36.6	0	0
15	36.3	0	-0.3
30	35.9	0	-0.7
45	35.7	0	-0.9
60	35.5	0	-1.1
75	35.3	0	-1.3
90	35.3	0	-1.3
105	35.1	0	-1.5
120	35	0	-1.6
135	34.9	0	-1.7
150	34.7	0	-1.9
165	34.6	0	-2
180	34.5	0	-2.1
195	34.3	0	-2.3
210	34.2	0	-2.4
225	34.1	0	-2.5
240	33.9	0	-2.7

pig 2	warmed insufflation group		
temps	tempera	volume	deltaT
0	35.8	0	0
15	35.6	30.6	-0.2
30	35.4	60.2	-0.4
45	35.3	91.1	-0.5
60	35.1	120	-0.7
75	35	150	-0.8
90	34.7	180	-1.1

pig 3	warmed insufflation group		
temps	tempera	volume	deltaT
0	39.4	0	0
15	39	30	-0.4
30	38.8	360	-0.6
45	38.7	90	-0.7
60	38.6	120	-0.8
75	38.5	1250	-0.9
90	38.4	180	-1

pig 4	warmed insufflation group		
temps	tempera	volume	deltaT
0	37	0	0
15	36.8	68.7	-0.2
30	36.5	93.5	-0.5
45	36.4	101	-0.6
60	36.2	119	-0.8
75	36.1	153	-0.9
90	36	180	-1

pig 1	warmed insufflation group		
temps	tempera	volume	deltaT
0	36	0	0
15	35.9	30	-0.1
30	35.9	60	-0.1
45	35.8	90	-0.2
60	35.8	120	-0.2
75	35.7	150	-0.3
90	35.6	180	-0.4

105	34.5	210	-1.3	105	38.4	210	-1	105	35.9	210	-1.1	105	35.5	210	-0.5
120	34.2	240	-1.6	120	38.4	240	-1	120	35.8	240	-1.2	120	35.5	240	-0.5
135	34	270	-1.8	135	38.3	270	-1.1	135	35.7	270	-1.3	135	35.4	270	-0.6
150	33.8	300	-2	150	38.3	300	-1.1	150	35.6	300	-1.4	150	35.2	300	-0.8
165	33.7	330	-2.1	165	38.2	330	-1.2	165	35.5	330	-1.5	165	35.2	330	-0.8
180	33.5	360	-2.3	180	38.2	360	-1.2	180	35.5	360	-1.5	180	35.1	360	-0.9
195	33.3	390	-2.5	195	38.2	390	-1.2	195	35.4	390	-1.6	195	35	390	-1
210	33.2	420	-2.6	210	38.2	420	-1.2	210	35.3	420	-1.7	210	35	420	-1
225	33	450	-2.8	225	38.2	450	-1.2	225	35.3	450	-1.7	225	34.9	450	-1.1
240	32.8	480	-3	240	38.3	480	-1.1	240	35.2	480	-1.8	240	34.8	480	-1.2

pig 1	forced air group			pig 2	forced air group			pig 3	forced air group			pig 4	forced air group		
temps	tempera	volume	deltaT	temps	tempera	volume	deltaT	temps	tempera	volume	deltaT	temps	tempera	volume	deltaT
0	36.8	0	0	0	37.9	0	0	0	37		0	0	35.9	0	0
15	36.7	30	-0.1	15	37.9	30	0	15	36.7		-0.3	15	35.5	30	-0.4
30	36.4	60.1	-0.4	30	37.9	60.1	0	30	36.5		-0.5	30	35.4	60	-0.5
45	36.2	90.2	-0.6	45	37.9	90.1	0	45	36.5		-0.5	45	35.2	90	-0.7
60	36	120	-0.8	60	37.8	120	-0.1	60	36.6		-0.4	60	35.1	120	-0.8
75	35.8	150	-1	75	37.8	150	-0.1	75	36.7		-0.3	75	34.9	150	-1
90	35.6	181	-1.2	90	37.9	180	0	90	36.7		-0.3	90	34.8	180	-1.1
105	35.5	210	-1.3	105	37.9	210	0	105	36.7		-0.3	105	34.7	210	-1.2
120	35.4	241	-1.4	120	37.9	240	0	120	36.7		-0.3	120	34.4	240	-1.5
135	35.3	270	-1.5	135	38	270	0.1	135	36.8		-0.2	135	34.4	270	-1.5
150	35.1	3300	-1.7	150	38	300	0.1	150	36.8		-0.2	150	34.2	300	-1.7
165	34.8	330	-2	165	38.1	330	0.2	165	36.8		-0.2	165	34.1	330	-1.8
180	34.6	360	-2.2	180	38.2	360	0.3	180	36.8		-0.2	180	33.9	360	-2
195	34.4	390	-2.4	195	38.2	390	0.3	195	36.9		-0.1	195	33.8	390	-2.1
210	34.2	420	-2.6	210	38.3	421	0.4	210	36.9		-0.1	210	33.6	420	-2.3
225	34	450	-2.8	225	38.3	450	0.4	225	36.9		-0.1	225	33.5	450	-2.4
240	33.5	480	-3.3	240	38.4	480	0.5	240	37		0	240	33.4	480	-2.5

pig 3	combination group		
temps	tempera	volume	deltaT
0	37	0	0
15	37.1	30	0.1
30	37.1	60	0.1
45	37.1	90	0.1
60	37.1	120	0.1
75	37	150	0
90	37	180	0
105	37	210	0
120	37	240	0
135	37	270	0
150	36.9	300	-0.1
165	36.8	330	-0.2
180	36.8	360	-0.2
195	36.8	390	-0.2
210	36.8	420	-0.2
225	36.7	450	-0.3
240	36.7	480	-0.3

pig 4	combination group		
temps	tempera	volume	deltaT
0	38.4	0	0
15	38.5	30	0.1
30	38.5	60	0.1
45	38.5	90	0.1
60	38.5	120	0.1
75	38.5	150	0.1
90	38.6	180	0.2
105	38.6	210	0.2
120	38.7	240	0.3
135	38.7	270	0.3
150	38.8	300	0.4
165	38.8	330	0.4
180	38.9	360	0.5
195	39	390	0.6
210	39.1	420	0.7
225	39.2	450	0.8
240	39.2	480	0.8

pig 1	combination group		
temps	tempera	volume	deltaT
0	37.9	0	0
15	37.3	15	-0.6
30	37.4	60.2	-0.5
45	37.4	90.2	-0.5
60	37.2	120	-0.7
75	37.2	150	-0.7
90	37.1	180	-0.8
105	37.1	210	-0.8
120	37.1	240	-0.8
135	37.2	270	-0.7
150	37.4	300	-0.5
165	37.5	330	-0.4
180	37.6	36	-0.3
195	37.7	390	-0.2
210	37.9	420	0
225	38	450	0.1
240	38.1	480	0.2

pig 2	combination group		
temps	tempera	volume	deltaT
0	36.1	0	0
15	36.1	30	0
30	36.1	60.1	0
45	36.1	90.2	0
60	36.2	120	0.1
75	36.2	150	0.1
90	36.3	180	0.2
105	36.3	210	0.2
120	36.4	242	0.3
135	36.4	271	0.3
150	36.5	301	0.4
165	36.5	331	0.4
180	36.6	360	0.5
195	36.6	390	0.5
210	36.6	420	0.5
225	36.7	449	0.6
240	36.7	480	0.6
